# Niacinamide enhances cathelicidin mediated SARS-CoV-2 membrane disruption

**DOI:** 10.3389/fimmu.2023.1255478

**Published:** 2023-11-08

**Authors:** Tanay Bhatt, Binita Dam, Sneha Uday Khedkar, Sahil Lall, Subhashini Pandey, Sunny Kataria, Johan Ajnabi, Shah-E-Jahan Gulzar, Paul M. Dias, Morris Waskar, Janhavi Raut, Varadharajan Sundaramurthy, Praveen Kumar Vemula, Naresh Ghatlia, Amitabha Majumdar, Colin Jamora

**Affiliations:** ^1^ IFOM-inStem Joint Research Laboratory, Centre for Inflammation and Tissue Homeostasis, Institute for Stem Cell Science and Regenerative Medicine (inStem), Bangalore, Karnataka, India; ^2^ Department of Biological Sciences, Manipal Academy of Higher Education (MAHE), Manipal, Karnataka, India; ^3^ National Centre for Biological Sciences (TIFR), Bangalore, Karnataka, India; ^4^ Integrative Chemical Biology, Institute for Stem Cell Science and Regenerative Medicine (inStem), Bangalore, Karnataka, India; ^5^ Unilever R&D, Bangalore, Karnataka, India; ^6^ Unilever R&D, Trumbull, CT, United States

**Keywords:** COVID-19, SARS-CoV-2, antimicrobial peptides, niacinamide, LL37, skin immunity, viral membrane

## Abstract

The continual emergence of SARS-CoV-2 variants threatens to compromise the effectiveness of worldwide vaccination programs, and highlights the need for complementary strategies for a sustainable containment plan. An effective approach is to mobilize the body’s own antimicrobial peptides (AMPs), to combat SARS-CoV-2 infection and propagation. We have found that human cathelicidin (LL37), an AMP found at epithelial barriers as well as in various bodily fluids, has the capacity to neutralise multiple strains of SARS-CoV-2. Biophysical and computational studies indicate that LL37’s mechanism of action is through the disruption of the viral membrane. This antiviral activity of LL37 is enhanced by the hydrotropic action of niacinamide, which may increase the bioavailability of the AMP. Interestingly, we observed an inverse correlation between LL37 levels and disease severity of COVID-19 positive patients, suggesting enhancement of AMP response as a potential therapeutic avenue to mitigate disease severity. The combination of niacinamide and LL37 is a potent antiviral formulation that targets viral membranes of various variants and can be an effective strategy to overcome vaccine escape.

## Introduction

**Figure 1:**
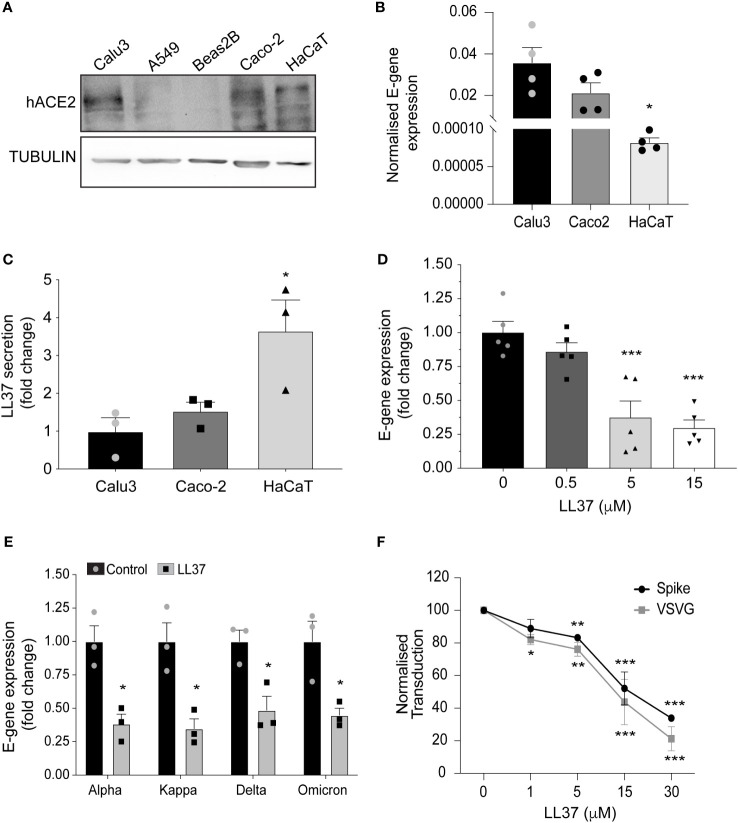
Antiviral activity of LL37 against SARS-CoV-2 variants **(A)** ACE2 protein levels in various epithelial cell lines (Western blotting) **(B)** SARS-CoV-2 E-gene expression in Calu3, Caco2, and HaCaT cells, 24 hr post infection (qRT-PCR) (n=4) **(C)** Secreted LL37 levels in Calu3, Caco2 and HaCaT cells (ELISA) (n=3) **(D)** Effect of increasing LL37 concentrations on SARS-CoV-2 neutralization (qRT-PCR) (n=5) **(E)** Effect of LL37 on the infectivity of various SARS-CoV-2 variants (qRT-PCR) (n=3) **(F)** Neutralization of Pseudo type virus (spike and VSVG) in the presence of LL37 (FACS) (n=3) [Statistical analysis was done using student's t-test **(B, E)** one-way ANOVA **(C, D)**, two-way ANOVA **(F)**,*p≤0.05, **p≤0.001, ***p≤0.0001].

SARS-CoV-2 infects host cells by the engagement of its spike protein with the angiotensin converting enzyme 2 (ACE2) receptor on the cell membrane (1). The importance of this interaction underlies the strategy of many vaccines to target Spike protein to disrupt this interaction and thus prevent infection (2). While exogenous ACE2 expression is sufficient to render cells competent for SARS-CoV-2 infection (3), tissue expression of ACE2 is not always an indicator of viral tropism (4). A case in point is the skin, which expresses ACE2 in the epidermis *in-vivo (* 5), and keratinocytes *in-vitro* (**Figures 1A**, **S1A**) but nevertheless, it is not considered as a primary route of infection (6). In support of this, exposure of human epidermal keratinocytes to SARS-CoV-2 does not result in a productive infection (**Figure 1B**). Thus, although epidermal keratinocytes are competent for SARS-CoV-2 infection, they may possess an endogenous defence mechanism to inhibit viral infection. The skin possesses a basal defence mechanism endowed by the constitutive secretion of antimicrobial peptides (AMPs) (7). In particular, the human AMP cathelicidin (LL37) has been shown to target various classes of viruses (8, 9), including respiratory viruses (10). A higher basal level of LL37 secretion in skin keratinocytes (**Figure 1C**) correlates with their lower infectivity by SARS-CoV-2 (**Figure 1B**).

Previous reports suggest LL37 as a potent inhibitor of SARS-CoV-2 through its interaction with ACE2 and Spike proteins (11). It has also been shown to be a potent antiviral molecule against many viruses, including influenza A virus (IAV), human immunodeficiency virus (HIV), Zika virus (ZIKV), and dengue virus (DENV-2) (8, 9, 12, 13). In addition to its direct antimicrobial activities, LL37 is also known to modulate the host immune system through its interactions with various cell surface receptors thereby generating a comprehensive host response against infection (14).

Despite the extensive evidence of LL37 as an antimicrobial, a limitation of its therapeutic application lies in the inherent property of this cationic peptide to self-aggregate, thereby limiting its bioavailability and efficacy (15, 16). To this end, many formulations are including hydrotropes to enhance the solubility and bioavailability of numerous biomolecules (17). One such hydrotrope is niacinamide (vitamin B3) which is widely used in various cosmeceutical and pharmaceutical products (18). Niacinamide has also been shown to impart various antimicrobial properties through the induction of antimicrobial peptide secretion from skin cells (19). Moreover, it has also been shown to enhance the activity of LL37 and its efficacy against bacterial cell membrane (20). Thus we hypothesized that the addition of niacinamide would enhance the efficacy of LL37 as an antiviral agent and probed the mechanism of action and its practical use against a respiratory virus.

## Results

To ascertain whether LL37 is effective against SARS-CoV-2, we incubated the virus with this AMP and assessed its ability to infect a reporter cell line, the intestinal epithelial cell (Caco-2). We observed a dose-dependent decrease in viral gene expression upon treatment with LL37 (**Figure 1D**). This LL37 mediated effect was also observed in other SARS-CoV-2 variants (alpha, kappa, delta, and omicron) (**Figure 1E**). These observations were confirmed by tissue-culture infectious dose (TCID50) assays (**Figure S1B**). Previous work has suggested that LL37 can interact with the spike protein and the ACE2 receptor and possibly occlude the interaction surface between them (11). However, LL37 has also been shown to interact with, and aggregate on membranes (21). Hence, we hypothesized that LL37 may inhibit viral infection in a Spike/ACE2 independent manner. We thus compared the neutralising capacity of LL37 against viruses with a different tropism, namely VSV-G and S1 pseudotyped lentivirus particles. We observed comparable reductions in viral transduction when both pseudotyped lentiviral particles were treated with increasing amounts of LL37 (**Figure 1F**).

**Figure 2:**
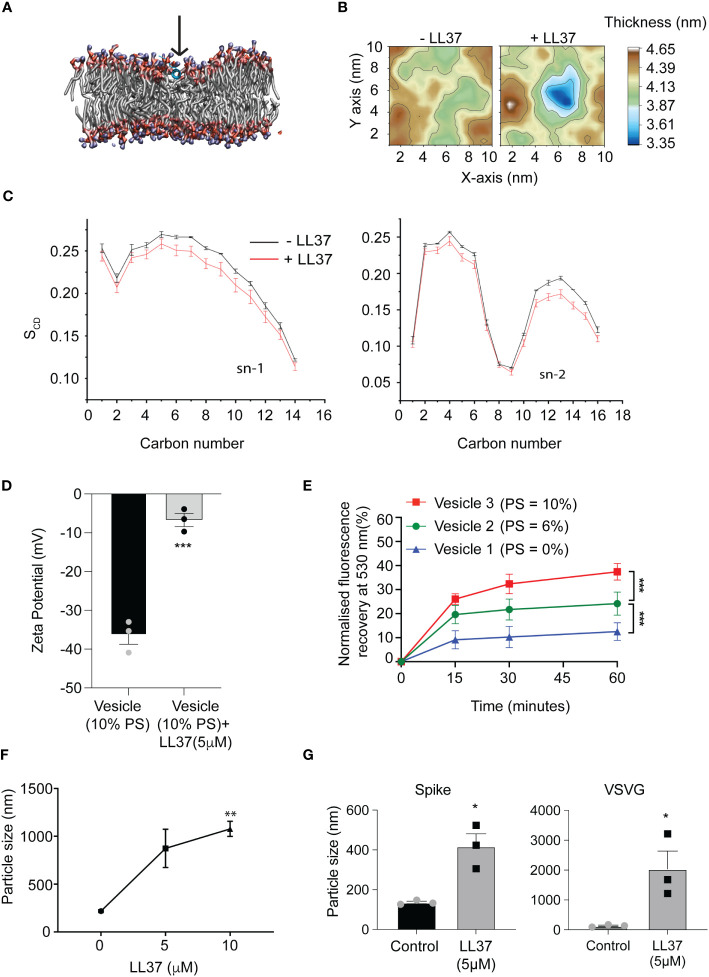
Mode of action of LL37 peptide mediating antiviral activity **(A)** The LL37 peptide gets deeply embedded in the membrane in 200ns **(B)** Representative membrane thickness averaged over the last 20 ns of MD simulations in absence (left) and presence (right) of the LL37 peptide. **(C)** LL37 causes a reduction in lipid ordering. Both the saturated (sn-1) and the unsaturated (sn-2) lipid acyl chains exhibit reduced ordering in presence of the LL37 peptide, quantified (bottom) using the lipid order parameter (S_CD_) (mean ± SD from last 20ns of 5 independent simulations) **(D)** Charge neutralization of vesicles in the presence of LL37 (n=3) **(E)** Membrane disruption assay using virus-like vesicles, mimicking viral membrane and PS concentration (FRET) (n=3) **(F)** Particle size analysis of SARS-CoV-2 in the presence of LL37 (DLS) (n=3) **(G)** Particle size analysis of Pseudovirus (Spike and VSVG) in the presence of LL37 (DLS) (n=3) [Statistical analysis was done using student t-test for **(D, G)**, two-way ANOVA for **(E)**, and one-way ANOVA(F) *p≤0.05, **p≤0.001, ***p≤0.0001].

These results are consistent with reports that LL37, a cationic peptide, can execute its antimicrobial activity by attacking the negatively charged membrane of pathogens (22). Moreover LL37 is known to adsorb onto lipid bilayers (23). To investigate the early steps of LL37 adsorption on viral envelope-like membranes, we employed molecular dynamics (MD) simulations. These simulations reveal that lipid acyl chains and headgroups interact with the LL37 peptide. In accordance with previous literature these interactions pull the peptide deeper into the membrane (**Figure 2A**), inducing local thinning of the bilayer (**Figure 2B**) and ultimately destabilizing the lipid order (**Figure 2C**). To experimentally verify the membrane adsorption and destabilization of the viral envelop, we prepared lipid vesicles to mimic the membrane composition of a generic coronavirus. Enveloped viruses such as coronavirus that assemble virions by budding off from the endoplasmic reticulum membrane have a negatively charged membrane due to a higher content of phosphatidylserine (PS) (24, 25). We prepared three different vesicles in which PS composition was varied according to the published range of a model coronavirus (25) (**Figure S1C**). We observed that increasing the percentage of PS resulted in an increase in the negative surface charge on the vesicles (**Figure S1D**), which was neutralized by the presence of LL37 (**Figure 2D**). These results suggest that the positively charged peptide can coat the outer leaflet of the bilayer likely by electrostatic interactions. To determine the consequence of the interaction of LL37 with the vesicles, we assayed whether membrane integrity was compromised. Using a fluorescence resonance energy transfer (FRET) based membrane disruption assay (schematically shown in **Figure S1E**), we observed reduction in FRET (fluorescence recovery at 530nm) when vesicles were treated with LL37 (**Figure 2E**). These results indicate that LL37 is more effective in interacting with and disrupting membranes with a higher negative charge (**Figures S1D**, **2E**). Previous reports have also indicated that disruption of vesicle membranes by positively charged polymers leads to vesicle clumping (26). We therefore investigated whether disruption of the pseudoviruses and SARS-CoV-2 by LL37 would result in their aggregation leading to increase in particle size as measured by dynamic light scattering (DLS). Consistent with the reported effect of cationic polymers on negatively charged membranes, we observed an increase in particle size of SARS-CoV-2 (**Figure 2F**) as well as pseudotyped virus (VSV-G and Spike) (**Figure 2G**) upon treatment with LL37.

**Figure 3:**
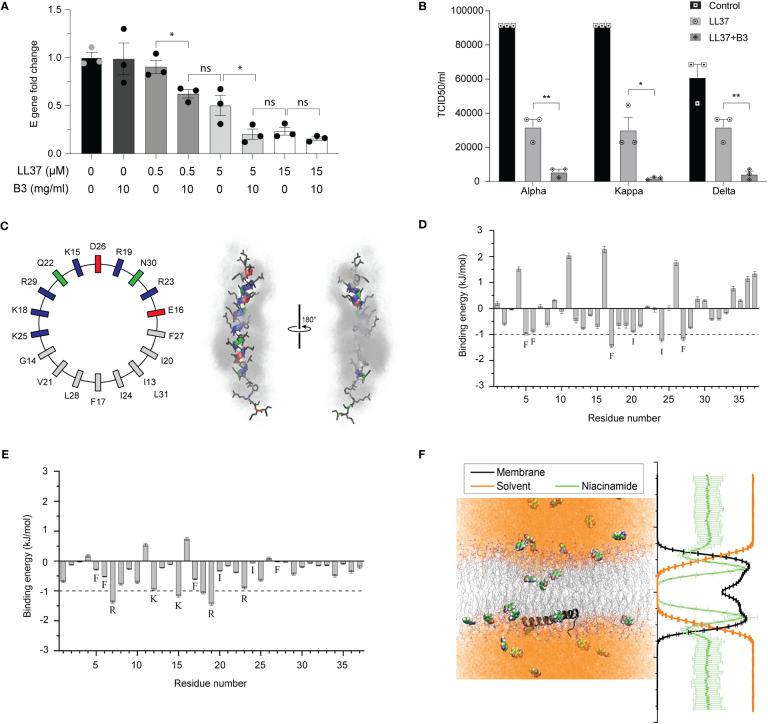
Effect of niacinamide on LL37 **(A)** Viral gene expression at different concentrations of LL37 in the presence of niacinamide (qRT-PCR) (n=3) **(B)** Effect of niacinamide combined with LL37 on the TCID50/ml of various SARS-CoV-2 strains (n=3) **(C)** Partial sequence of the amphipathic LL37 peptide is represented with an idealized helical wheel (left) with residues colored according to their nature (hydrophobic, grey; polar, green; acidic, red; basic, blue). Right, two views of the LL37 peptide (color scheme same as the wheel; sidechains, black) overlaid with niacinamide molecules (grey) within 6 Å of the peptide (over 10 ns). The 10 ns overlay emphasizes the solvent-like encapsulation of the peptide by niacinamide with greater residence over the hydrophobic face of the helix than the polar/charged face **(D)** Contribution of each residue of LL37 towards interaction with niacinamide from MD simulations (mean of 3 independent simulations, errors propagated from each replicate). Residues contributing >1 kJ mol-1 are labelled **(E)** Niacinamide in the membrane forms hydrogen bonds with the Lys (K) and Arg (R) residues of LL37. Whereas, the aliphatic residues of LL37 which preferentially interacted with niacinamide in solution **(D)** are now predominantly engaged with the hydrophobic acyl chains and so, are unable to contribute much to bind niacinamide **(F)** Representative snapshot (left) of niacinamide penetrating deeper than water into the hydrophobic core of the membrane (LL37, black peptide; lipid acyl chains, grey lines; niacinamide, space filling; water, orange) which is quantified on the right, averaging the molecular density (membrane, black; niacinamide, green; water, orange) over the entire 200 ns trajectory (5 replicates, mean ± SD) [Statistical analysis was done using student t-test for **(A)** and two-way ANOVA for **(B)**, *p≤0.05, **p≤0.001, ***p≤0.0001].

Previous reports have indicated that self-aggregation of soluble LL37 molecules decreases its bioavailability and hinders its antimicrobial activity. Thus, it has been speculated that decreasing the AMP’s inherent self-aggregation (21) could improve its bioavailability and efficacy against viruses such as SARS-CoV-2. A common approach to prevent aggregation is with the use of a hydrotrope. Niacinamide (vitamin B3) is a generally regarded as safe (GRAS) hydrotrope used to increase the solubility, and therefore the activity, of various drugs (18). Indeed, we observed that LL37 supplemented with niacinamide exhibited an enhanced potency against infection by different variants of SARS-CoV-2 (**Figures 3A, B**), while niacinamide alone was not antiviral (**Figure 3A**). To understand the mechanism of how LL37 interacts with lipid membranes in the presence of niacinamide, we performed atomistic MD simulations of LL37 in a solution containing niacinamide. The hydrophobic phenylalanine (Phe5, Phe6, Phe17 and Phe27) and isoleucine (Ile 20 and Ile 24) residues, which mediate LL37’s self-aggregation and reduce its activity, preferentially interacted with niacinamide molecules over water molecules (**Figures 3C, D**). These aromatic-π and van der Waals interactions of the hydrophobic residues of LL37 with niacinamide would hydrotropically solubilise the LL37 peptide (17). This encapsulation of aggregation prone residues of the AMP LL37 would improve the solubility and bioavailability of the peptide which can improve its membrane disrupting activity. In-silico analysis suggests that LL37 interacts with the membrane along its hydrophobic face, freeing the charged amino acid residues to interact with the solvent, which in this case is the niacinamide solution (**Figure 3E**). Additionally, these simulations demonstrate that niacinamide can also embed deeply into the membrane (**Figure 3F**) and may synergize with LL37 to disrupt the lipid bilayer. Thus, we conclude that niacinamide has a dual effect: (i) hydrotropic increase of the aqueous solubility of LL37, thereby rendering it more bioavailable and; (ii) cooperation with the AMP to destabilize membranes.

**Figure 4:**
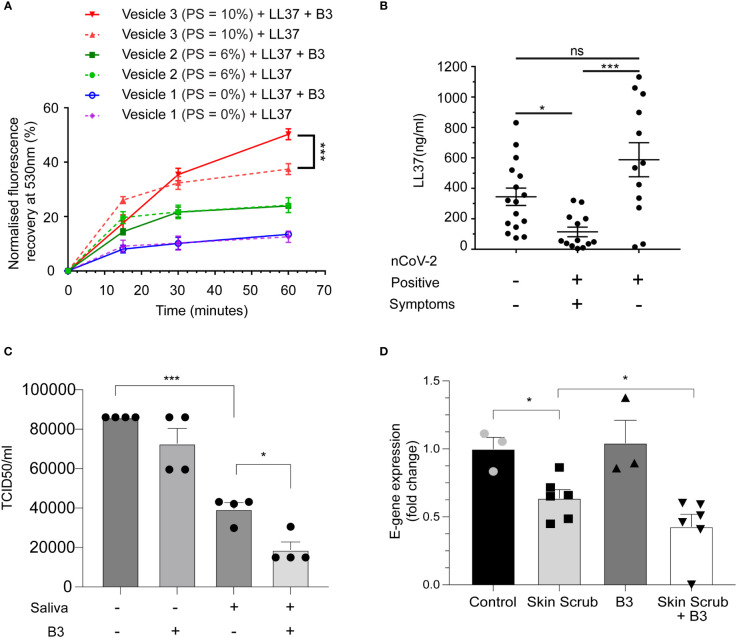
Niacinamide enhances antiviral activity of LL37 **(A)** Membrane disruption assay on addition of LL37 and niacinamide (FRET) (n=3) **(B)** Measurement of LL37 in various patient cohorts (ELISA) (A total of 41 individuals were subdivided into negative (16), positive symptomatic (13), and positive asymptomatic (12) cohorts **(C)** Effect of niacinamide addition to saliva on SARS-CoV-2 neutralisation (TCID50) (n=4) **(D)** Effect of skin scrub and its combination with niacinamide on SARS-CoV-2 viral gene expression (RT-qPCR) (Control, B3: n=3, Skin scrub, Skin scrub + B3: n=6).[Statistical analysis was done using two-way ANOVA for **(A)**, Kruskal Wallis test (non-parametric ANOVA) for **(B)**, one-way ANOVA for **(C)**, and student t-test for **(D)**].

To validate these computational results, we performed the same FRET-based membrane disruption assay using artificial viral membranes (as described in **Figures S1C, D**) but this time in the presence of LL37 and niacinamide. Consistent with predictions from the MD simulations, we observed that membrane disruption of liposomes by LL37 was enhanced in combination with niacinamide (**Figure 4A**).

Disease severity of several viral respiratory infections has been inversely correlated with LL37 levels (27). Since it has been shown that salivary burden of SARS-CoV-2 correlates with disease severity in patients (28), we compared the levels of secreted LL37 in the saliva of SARS-CoV-2 infected and uninfected individuals (patient information in **Figure S1F**). Symptomatic individuals had on average ~3-fold less LL37 than uninfected individuals (**Figure 4B**). Interestingly, asymptomatic positive patients had equivalent levels of LL37 as uninfected individuals. These results suggest that lower LL37 levels may potentially render individuals more susceptible to a symptomatic infection. Since, lower levels of LL37 is associated with the symptomatic COVID-19 patient group (**Figure 4B**), supplementation of enhancers like, B3, which can enhance the activity of existing LL37 can serve as an excellent therapeutic option. To test whether the effect of niacinamide can be reproduced with naturally produced AMPs, we analyzed its effect on AMPs that are highly secreted in saliva (29) and from the skin (7). We found that human saliva exhibits antiviral activity against SARS-CoV-2, which can be potentiated upon supplementation with niacinamide (**Figure 4C**). Likewise, we also observed that skin scrubs supplemented with niacinamide exhibited antiviral activity (**Figure 4D**). Our body naturally synthesizes niacinamide, but interestingly, the biosynthetic pathways and precursor leading to niacinamide production are downregulated in symptomatic COVID-19 patients (30). Thus, exogenous supplementation of niacinamide in symptomatic patients may potentiate the activity of naturally produced AMPs from the body’s epithelia.

## Discussion

The variants of SARS-CoV-2 that exhibit increased transmissibility and disease severity are reported to contain mutations in the receptor binding domain (RBD) of the spike protein (31). Given their key role in mediating viral entry into the host cell, many vaccines have been developed with antigens derived from the spike protein, and mutations pose a serious problem with vaccine escape (32). In addition, the heavy glycan coating of the spike proteins can be a mechanism of camouflaging them from the immune system (33). One approach to circumvent these problems is to target the viral envelope that originates from the host cell and is thus conserved among the different variants. Altogether, we show that the AMP LL37 has the potential to neutralise the SARS-CoV-2 viral infection by targeting its envelope, and niacinamide further enhances this antiviral activity of the peptide. Our data on the symptomatic patient samples further substantiate this hypothesis and argues for an approach that would entail enhancing the efficacy of antimicrobial peptides for protection against viral infections. Therefore, either exogenous administration of the AMP with niacinamide or other strategies to boost the endogenous production of the peptide in combination with niacinamide could be a potent method to not only block viral transmission, but may be an effective therapy to limit viral load and disease severity of a patient post infection. Therefore, the next step in this program would be to develop a delivery system of the LL-37 and niacinamide formulation directly in the respiratory tract. One possible approach that is currently being used for the delivery of vaccines is through nebulizers or nasal sprays. Animal models of SARS-CoV-2 infection would thus be useful for the development of this therapeutic modality.

## Materials and methods

### Cell lines and reagents

Caco-2 (ATCC HTB-37), Vero-E6 (ATCC CRL-1586), Calu3 (ATCC HTB-55) and HEK 293T cells (ATCC CRL-3216) were grown in Dulbecco’s Modified Eagle’s Medium (DMEM, Gibco), with 10% fetal bovine serum (FBS, Gibco), 100U/ml penicillin and 100µg/ml streptomycin (Gibco) at 37° C in 5% CO2 incubator. Human keratinocyte cell line (HaCaT cells) were grown and differentiated as described elsewhere (34). All the experiments and incubations were carried out in serum free media such as OptiMEM (Gibco) or EpiLife (Gibco).

### Plasmids

Lentivirus packaging plasmid psPAX2 (Addgene #12260) and pMD2.G (Addgene #12259) were used for VSV-G pseudovirus production. For generating SARS-CoV-2 spike pseudotyped lentivirus, pTwist-EF1alpha-SARS-CoV-2-S-2xStrep (Addgene #141382) (35) was used. pZip-mEF1a-ZsGreen-Puro was used for the ZsGreen expression.

### Transfection and pseudotype virus production

293T cells were transfected with psPAX2, pZIP-mEF1a-ZSGreen-Puro, and either pMD2.G or pTwist-EF1alpha-SARS-CoV-2-S-2xStrep using LTX (Invitrogen) transfection reagents according to the manufacturer’s protocol. Following a 48 hr transfection, the virus particle-containing media was harvested and virus particles were purified using Polyethylene glycol 8000 (Sigma).

### Transduction and FACS

About 0.1 to 0.2 MOI (Multiplicity of Infection) equivalent of VSV-G and pseudotype SARS-CoV-2 spike virus particles were treated with recombinant LL37 (Tocris) and Vitamin B3 (provided by Unilever), both solubilised in sterile distilled water, for three hours at 37°C (in 100 uL volume). Treated lentivirus was then added to Caco-2 cells (96 well plate with about 30,000 cells/well) for transduction. ZsGreen expression was monitored after 72 hr of transduction. The cells were trypsinized and single cell suspension was analysed in an Attune NxT flow cytometer with appropriate gates to determine percentage of transduction in each sample.

### Dynamic light scattering analysis

Particle Size Analyser: Litesizer 500 model with a scattering angle of 90° angle was used to measure the particle size of pseudovirus particles (VSV-G and SARS-CoV-2 spike). The particles were treated with LL37, Vitamin B3 or their combination for one hour at 37°C in 100 uL volume. The samples were then diluted to 1 ml with Epilife media (Cat No. MEPI500CA). Readings were taken in a quartz cuvette at 25°C in series for triplicates. The measured data were processed by Kalliope™ software and intensity distribution curve was generated with peaks on the y-axis corresponding to particle intensity and their respective size (in nm) represented on the x-axis.

### Preparation and biophysical characterization of liposomes

Vesicle 1 was prepared with molar ratio of 83% DPPC (Dipalmitoyl phosphatidylcholine), 11% DOPE (Dioleoyl phosphatidylcholine), 6% DOPS (1,2-dioleoyl-sn-glycero-3-phospho-L-serine), Vesicle 2 (79% DPPC. 11% DOPE and 10%DOPS) and Vesicle 3 (DPPC: DOPE : DOPS: 85%: 15%: 00%) respectively, at a total concentration of 0.5mM. Dry thin films of lipids were prepared in glass vials, followed by overnight hydration with deionized water to produce hydrated films. Vortexing vials for 2-3 min produced multilamellar vesicles. Subsequently, bath sonication (Qsonica sonicator, Q700) produced small unilamellar vesicles.

### Zeta potential measurement

The vesicles’ sizes and zeta potentials (surface charges) were measured by photon correlation spectroscopy and electrophoretic mobility on a Litesizer 500, Anton Paar. The sizes and potentials of vesicles were measured in deionized water with a sample refractive index of 1.32 and a viscosity of 0.6912cP. The diameters of liposomes were calculated by using the automatic mode. The zeta potential was measured using the following parameters: dielectric constant, 74.19, 1.50 (Smoluchowski); maximum voltage of the current 100V. All the measurements were taken in triplicates.

### FRET assay for membrane disruption

Vesicles 1, 2, and 3 with a concentration of 0.5mM was prepared with FRET pair. Lipids NBD-PE and N-Rho-PE (Avanti-Polar Lipids, USA) were used as the donor and acceptor fluorescent lipids, respectively, with 0.005 mM NBD-PE and N-Rho-PE lipids (i.e., 1% with respect to the vesicle formulation content). Labelled vesicle formulations were placed in a fluorimeter, Microplate Fluorescence Reader (Horiba Instruments, USA) at 25 °C and LL37 (5µM) was added in the one set of experiments. In another set of experiments, both Vitamin B3(10mg/ml) and LL37(5µM) were added to labelled vesicles., Fluorescence intensities were recorded as a function of time with excitation at 485 nm and emission at 530 nm. Fusion (100%) was determined from the NBD-PE fluorescence intensity of the labelled vesicles formulation in the presence of 1% Triton X100 (experimental model in **Figure S1H**).

### SARS-CoV-2 infection

Experiments were carried out with 2019-nCoV/Italy-INMI1 strain unless otherwise stated to have used alpha, kappa, delta, and omicron strains of SARS-CoV-2. All the virus-related experiments were carried out in the BLiSc biosafety level 3 (BSL-3) laboratory. Virus particles, equivalent to 0.1 MOI, were incubated with recombinant LL37 and Vitamin B3 or buffer control for three hours in 100 μl volume. The pre-treated particles were then added to the host cells in a 24 well plate with about 240,000 cells/well and allowed to adsorb for one hour. The cells were then washed and replenished with fresh media.

### RNA isolation and qPCR

24 hr after incubation of pre-treated virus particles on cells, RNA was isolated using RNAiso Plus (Takara). 1 μg of RNA was used to prepare cDNA using the PrimeScript kit (Takara). cDNA equivalent to 100 ng of RNA was used for setting up the qPCR reaction using SYBR green master mix (Applied Biosystems). PCR reactions were performed using the CFX384 Touch Real-time PCR detection system (BioRad). All gene expression changes were calculated following normalization to βactin using the comparative Ct (cycle threshold) method. Quantitative real time PCR primers are listed here:

**Table:** 

Gene	Forward Primer (5'-3')	Reverse primer (5'-3')
LL37	TGACTTCAAGAAGGACGGGC	CAGGGCAAATCTCTTGTTATCCTTA
Beta-actin	TCCTTCCTGGGCATGGAGT	AGCACTGTGTTGGCGTACAG
E-gene	ACAGGTACGTTAATAGTTAATAGCGT	ATATTGCAGCAGTACGCACACA

### ELISA

Conditioned media of Calu3 and differentiated HaCaT were collected from 72 hr old confluent culture plates. The media were centrifuged at 10,000 x g for 3 minutes and used for ELISA. Unstimulated saliva samples were collected from uninfected donors. The samples were cleared by centrifugation at 10,000 x g for 5 minutes and the concentration of LL37 was determined by ELISA using a commercially available analysis kit specific for LL37 (Hycult Biotech, HK321-02), following manufacturer’s protocol. The absorbance was measured at 450 nm using a microplate reader (Varioskan, Thermo).

### TCID50

A median tissue culture infectious dose (TCID50) assay was performed to identify the viral titer of SARS-CoV-2 in control and treated conditions. VeroE6 cells were cultured in a 96-well tissue culture plate and varying dilutions of the virus were added. The virus was allowed to adsorb for 1 hr, following which cells were thoroughly washed and media was replenished. After incubation for 72 hr, the percentage of infected wells was observed for each dilution. These results were used to calculate the TCID50 value using a TCID50 calculator (by Marco Binder; adapted at TWC).

### Molecular dynamics simulations

Unrestrained all atom molecular dynamics (MD) simulations were performed on two different systems. The first system comprised one copy of LL37 (PDB id: 2K6O), 40 molecules of niacinamide (3NAP from CGenFF (36)) in a water solvated box of 1000 nm^3^ with 150 mM NaCl (**Figures 3C, D**). This system was simulated in triplicates of 50ns each. This starting configuration was created to approximate the experimental conditions with 0.5 µM peptide in a 10 mg/mL (70 mM) aqueous solution of niacinamide.

The other simulation was composed of a symmetric coronavirus lipid bilayer (25) comprising POPC : POPE : POPS : Cholesterol in a 70:15:10:5 ratio. One copy of LL37 peptide (2K6O) was adsorbed on the surface of the membrane using the initial configuration from the PPM server (37) and 40 molecules of niacinamide (3NAP, CGenFF) were added in the box of 10 x 10 x 14.5 nm^3^ (**Figures 2A–C**, **3E, F**). This was solvated and sodium counter ions were added to neutralize the charge. This configuration of 138,332 atoms was simulated in 5 replicates for 200 ns each. As a control, a fully solvated identical membrane was simulated in duplicate for 100ns each. All the particles were described by the CHARMM36 force field (38) and the simulations were performed on Gromacs (39) 2018. Inbuilt gromacs routines and g_mmpbsa (40) were used to analyze the simulations. Area per lipid and membrane thickness were estimated using Gridmat (41) and the lipid order was determined from equation 1 as implemented in Membrainy (42).


(1) $$S_{CD}=\;\langle \frac{3\cos^{2}\theta -1}{2}\rangle$$

### Saliva and skin scrub treatment of SARS-CoV-2

Virus particles equivalent to 0.1MOI were incubated with 100µl of either skin-scrub or control buffer at 37°C for four hours. Following this, the pre-treated particles were added on VeroE6 cells for TCID50 assay, or Caco-2 cells for qPCR and allowed to adsorb for one hour at 37°C. The cells were then washed and replenished with fresh media.

## Data availability statement

The original contributions presented in the study are included in the article/**Supplementary Material**. Further inquiries can be directed to the corresponding authors.

## Ethics statement

The studies involving human samples were approved by Institutional Biosafety Committee (inStem) Institutional Ethics Committee for Human Studies (inStem). The studies were conducted in accordance with the local legislation and institutional requirements. The human samples used in this study were acquired from the BLiSC COVID testing laboratory and stored in the BLiSC COVID biorespository. Written informed consent for participation was not required from the participants or the participants’ legal guardians/next of kin in accordance with the national legislation and institutional requirements.

## Author contributions

TB: Conceptualization, Formal Analysis, Writing – original draft, Writing – review & editing, Data curation, Investigation, Methodology, Validation. BD: Investigation, Writing – original draft, Writing – review & editing. SKh: Investigation, Methodology, Writing – original draft, Writing – review & editing. SL: Data curation, Investigation, Methodology, Formal analysis, Writing – original draft, Writing – review & editing. SP: Investigation, Methodology, Resources, Writing – review & editing. SKa: Investigation, Methodology, Writing – original draft. JA: Investigation, Methodology, Resources, Writing – review & editing. S-E-JG: Investigation, Methodology, Conceptualization, Writing – review & editing. PD: Conceptualization, Methodology, Resources, Writing – review & editing. MW: Conceptualization, Methodology, Resources, Writing – review & editing. JR: Conceptualization, Methodology, Resources, Writing – review & editing. VS: Methodology, Resources, Writing – review & editing. PV: Conceptualization, Investigation, Methodology, Resources, Writing – review & editing. NG: Conceptualization, Investigation, Writing – review & editing. AM: Funding acquisition, Writing – original draft, Writing – review & editing. CJ: Conceptualization, Formal Analysis, Funding acquisition, Project administration, Resources, Supervision, Writing – original draft, Writing – review & editing.

## Supplementary material

SUPPLEMENTARY FIGURE S1
**(A)** Densitometric quantification of western blot **Figure 1A** using image-J, normalised with beta Tubulin **(B)** Effect of LL37 on the TCID50/ml of various SARS-CoV-2 strains (n=3). **(C)** Membrane composition of the virus like vesicles **(D)** Size and charge of virus like vesicles **(E)** Principle and methodology of FRET based membrane disruption assay **(F)** Details of the patient group considered for this study [Statistical analysis was done using student’s t-test **(B)**, *p≤0.05, **p≤0.001, ***p≤0.0001].
