# Supplementary figures and images for: Niacinamide enhances cathelicidin mediated SARS-CoV-2 membrane disruption
Source: Front Immunol. 2023 Nov 8;14:1255478. doi: 10.3389/fimmu.2023.1255478 (PMC10663372; doi:10.3389/fimmu.2023.1255478)

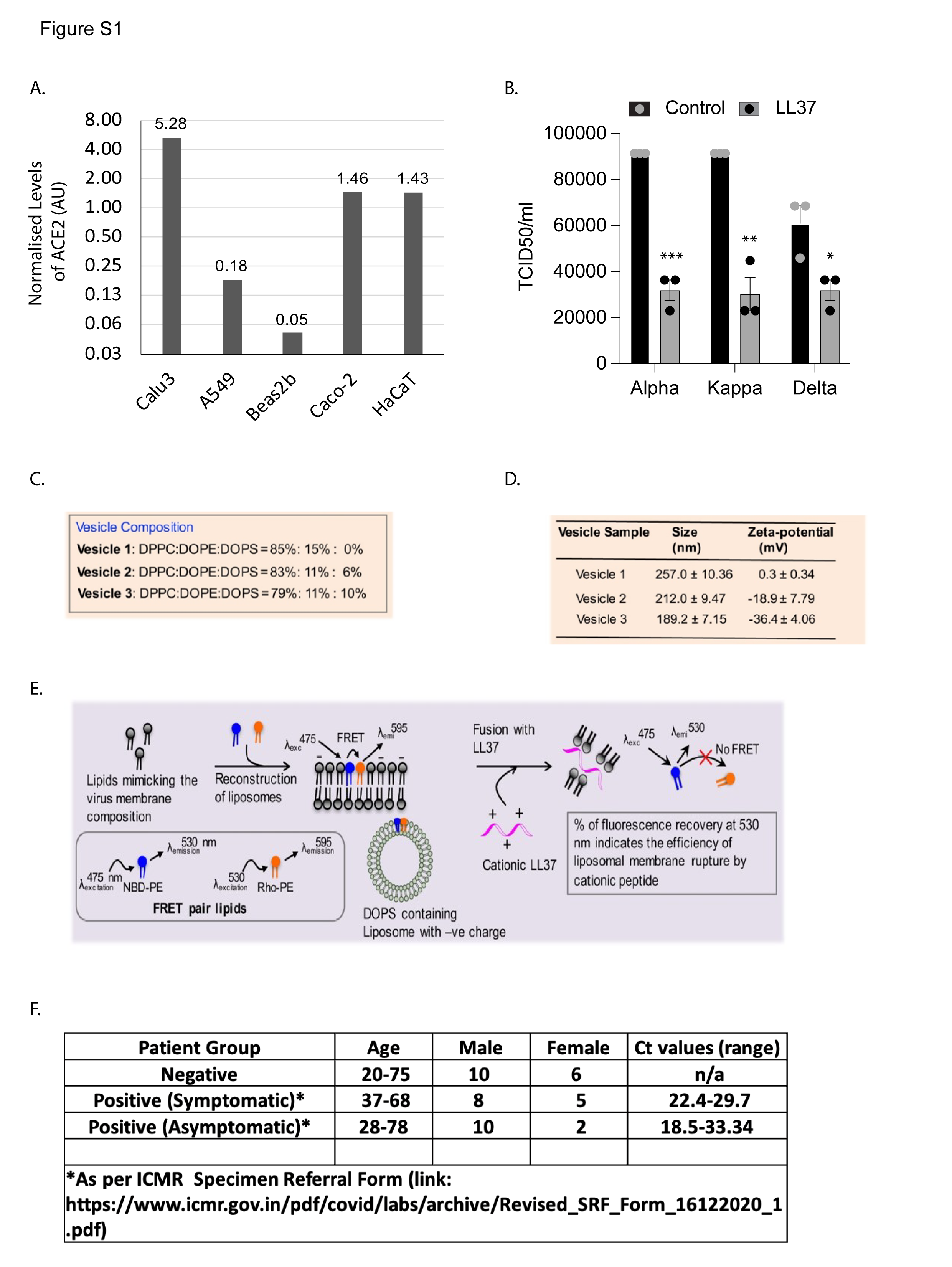

Supplement: SUPPLEMENTARY FIGURE S1 — (A) Densitometric quantification of western blot Figure 1A using image-J, normalised with beta Tubulin (B) Effect of LL37 on the TCID50/ml of various SARS-CoV-2 strains (n=3). (C) Membrane composition of the virus like vesicles (D) Size and charge of virus like vesicles (E) Principle and methodology of FRET based membrane disruption assay (F) Details of the patient group considered for this study [Statistical analysis was done using student’s t-test (B), *p≤0.05, **p≤0.001, ***p≤0.0001]. [file Image_1.tif]
